# Benchmarking and Analysis of Protein Docking Performance in Rosetta v3.2

**DOI:** 10.1371/journal.pone.0022477

**Published:** 2011-08-02

**Authors:** Sidhartha Chaudhury, Monica Berrondo, Brian D. Weitzner, Pravin Muthu, Hannah Bergman, Jeffrey J. Gray

**Affiliations:** 1 Program in Molecular Biophysics, Johns Hopkins University, Baltimore, Maryland, United States of America; 2 Department of Chemical and Biomolecular Engineering, Johns Hopkins University, Baltimore, Maryland, United States of America; University of South Florida, United States of America

## Abstract

RosettaDock has been increasingly used in protein docking and design strategies in order to predict the structure of protein-protein interfaces. Here we test capabilities of RosettaDock 3.2, part of the newly developed Rosetta v3.2 modeling suite, against Docking Benchmark 3.0, and compare it with RosettaDock v2.3, the latest version of the previous Rosetta software package. The benchmark contains a diverse set of 116 docking targets including 22 antibody-antigen complexes, 33 enzyme-inhibitor complexes, and 60 ‘other’ complexes. These targets were further classified by expected docking difficulty into 84 rigid-body targets, 17 medium targets, and 14 difficult targets. We carried out local docking perturbations for each target, using the unbound structures when available, in both RosettaDock v2.3 and v3.2. Overall the performances of RosettaDock v2.3 and v3.2 were similar. RosettaDock v3.2 achieved 56 docking funnels, compared to 49 in v2.3. A breakdown of docking performance by protein complex type shows that RosettaDock v3.2 achieved docking funnels for 63% of antibody-antigen targets, 62% of enzyme-inhibitor targets, and 35% of ‘other’ targets. In terms of docking difficulty, RosettaDock v3.2 achieved funnels for 58% of rigid-body targets, 30% of medium targets, and 14% of difficult targets. For targets that failed, we carry out additional analyses to identify the cause of failure, which showed that binding-induced backbone conformation changes account for a majority of failures. We also present a bootstrap statistical analysis that quantifies the reliability of the stochastic docking results. Finally, we demonstrate the additional functionality available in RosettaDock v3.2 by incorporating small-molecules and non-protein co-factors in docking of a smaller target set. This study marks the most extensive benchmarking of the RosettaDock module to date and establishes a baseline for future research in protein interface modeling and structure prediction.

## Introduction

The formation of highly specific protein complexes is a fundamental process in biology, and the structures of these complexes can yield deep insight into the mechanisms of protein function. Computational protein docking provides a means by which to predict the structure of protein-protein complexes from their unbound structures. Blind structure-prediction efforts, such as the Critical Assessment of Protein Interactions (CAPRI) [1], [2] have showcased a number of successful docking strategies using a range of methods from course-grained fast-Fourier transform approaches which identify surface complementarity between two partners [3], [4] to all-atom stochastic methods that can accommodate intricate protein conformational changes [5], [6]. In a number of CAPRI strategies, [3], [7], [8], [9], [10] as well as other protein docking studies [11], [12], the protein docking component of the Rosetta v2 software package, RosettaDock [13], has proved useful for a range of protein docking applications.

RosettaDock was first introduced as a multi-scale Monte Carlo based docking algorithm that utilized a centroid-based coarse grain stage to quickly identify favorable docking poses and an all-atom refinement stage that simultaneously optimized rigid-body position and side-chain conformation. Since then RosettaDock has been modified to address the critical challenge in protein-protein docking: binding-induced backbone conformational changes. Wang et al. introduced explicit loop modeling and backbone minimization [6] while we added ensemble-based docking [14] and conformational move sets specific to antibody docking [15]. In that span, RosettaDock has been used for a wide range of applications from antibody-antigen docking [11], [12], to peptide docking and specificity [16], [17] to multi-body [18] and symmetric docking.[19]

The current version of Rosetta, v3.2, has been in development for the past two years. The original Rosetta software package was written primarily for *ab initio* protein folding [20] but quickly expanded to include an array of molecular modeling applications from protein docking to enzyme design. The new Rosetta software package [21] was written from the ground up with these diverse applications in mind. Essential components such as energy function calculators, protein structure objects, and chemical parameters were assembled into common software layers accessible to all protocols. Protocols such as side-chain packing, or energy minimization, were written with a modular object-oriented architecture that allows users and programmers to easily combine different molecular modeling objects and functions. Control objects were written to give users a generalized scheme from which to precisely specify the sampling strategy for a given protocol. Finally, user interfaces such as RosettaScripts,[22] PyRosetta [23], and a PyMol interface [24] were developed to provide unprecedented accessibility of the code.

The protein docking component of Rosetta v3.2, was written with two main goals. The first goal was to include all the core docking capabilities of Rosetta v2.3. The second, take advantage of the modular Rosetta v3.2 architecture to easily include new features such as modeling small-molecules, [25] noncanonical amino acids, and post-translational modifications, adding more customized conformational constraints, or allowing for alternative side-chain packing or design schemes. In order to systematically evaluate docking performance, we ran both RosettaDock v2.3 and RosettaDock v3.2 against the recently expanded Protein Docking Benchmark 3.0 [26]. The results of this benchmark can determine whether RosettaDock v3.2 successfully reproduces or improves upon the results of RosettaDock v2.3. More importantly, benchmarking identifies the strengths and weakness of the core RosettaDock algorithm against a large diverse set of targets to guide future development.

Finally, in order to showcase the additional capabilities of the Rosetta v3 software package, we identified a subset of targets in the benchmark that contain small-molecule co-factors in or near the binding site. Although these co-factors are critical to biological protein function and interactions, due to their non-protein nature, they are often excluded from many docking algorithms, including Rosetta v2.3. We utilize the small-molecule modeling components of Rosetta v3.2 to incorporate these co-factors in the docking process to test whether performance would improve.

## Methods

### Overview of the RosettaDock algorithm

**Figure 1:**
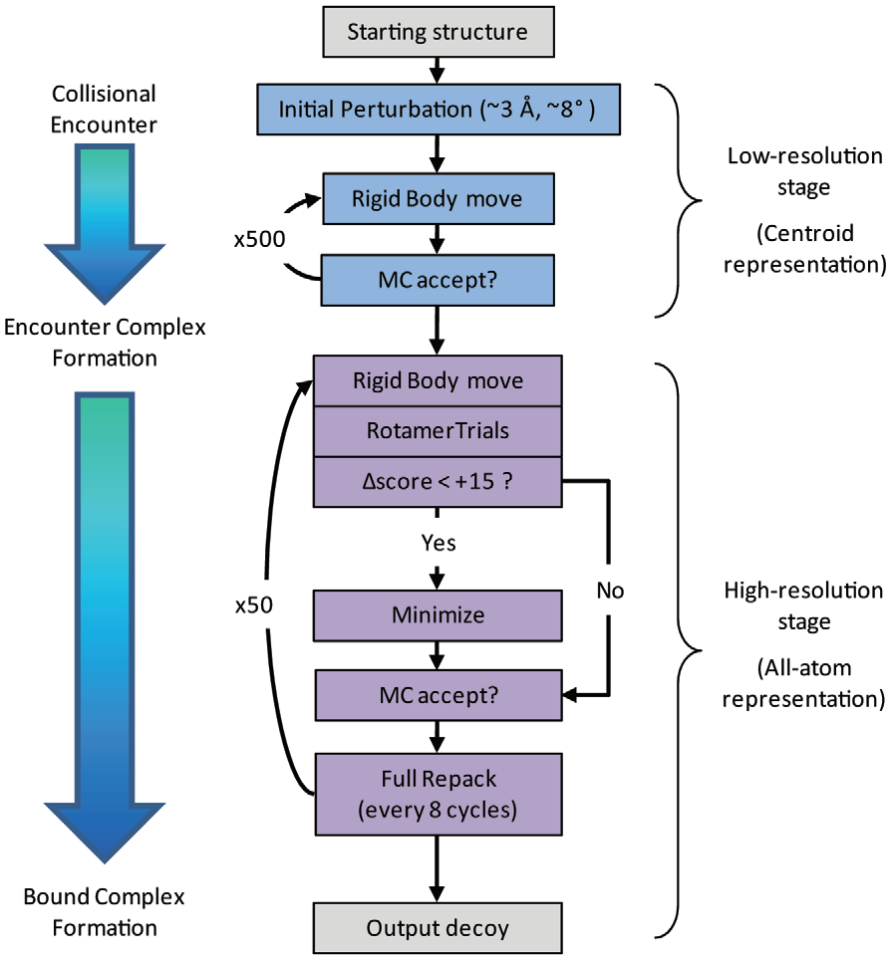
The RosettaDock algorithm. RosettaDock is a multi-scale Monte-Carlo based algorithm that roughly models encounter complex formation followed by a transition to a bound state.

RosettaDock is a Monte Carlo (MC) based multi-scale docking algorithm that incorporates both a low-resolution, centroid-mode, coarse-grain stage and a high-resolution, all-atom refinement stage that optimizes both rigid-body orientation and side-chain conformation. The algorithm, illustrated in Figure 1, roughly follows the biophysical theory of an encounter complex formation followed by a transition to a bound state. Typically the algorithm starts from either a random initial orientation of the two partners (global docking), or an initial orientation that is randomly perturbed from a user-defined starting pose (local perturbation). From there, the partner proteins are represented coarsely, where side chains are replaced by a single unified pseudo-atom, or centroid. During this phase, a 500-step Monte Carlo search is made with adaptive rotation and translational steps adjusted dynamically to achieve an acceptance rate of 25%. The score function used in this stage primarily consists of a ‘bump’ term, a contact term, and docking-specific statistical residue environment and residue-residue pair-wise potentials (Table 1) [13].

**Table 1:** RosettaDock scoring components and weights.

*Centroid docking score function*		
Component	name	weight
Contact	interchain_contact	2.0
Bumps	interchain_vdw	1.0
Environment	interchain_env	1.0
Pair-wise interaction	interchain_pair	1.0

*All-atom docking score function*		
Component	name	weight
Van der Waals (attractive)	fa_atr	0.338
Van der Waals (repulsive)	fa_rep	0.044
Dunbrack side-chain	fa_dun	0.036
Solvation	fa_sol	0.242
Pair-wise interaction	fa_pair	0.164
Hydrogen Bond	hbond_lr_bb, hbond_sr_bb, hbond_bb_sc, hbond_sc	0.245
Electrostatic	hack_elec	0.026

Once the centroid-mode stage is complete, the lowest energy structure accessed during that stage is selected for high-resolution refinement. During high-resolution refinement, centroid pseudo-atoms are replaced with the side-chain atoms at their initial unbound conformations. Then 50 MC steps are made in which the (1) rigid-body position is perturbed by a random direction and magnitude specified by a Gaussian distribution around 0.1 Å and 3.0°, (2) the rigid-body orientation is energy-minimized, and (3) side-chain conformations are optimized with RotamerTrials [27], followed by a test of the Metropolis criteria. Every eight steps, an additional combinatorial side-chain optimization is carried out using the full side-chain packing algorithm, followed by an additional Metropolis criteria check. To reduce the time devoted to the computationally expensive energy-minimization for unproductive rigid-body moves, minimization is skipped if a rigid-body move results in a change in score of greater than +15. The all-atom score function used in this stage primarily consists of Van der Waals attractive and repulsive terms, a solvation term, an explicit hydrogen bonding term, a statistical residue-residue pair-wise interaction term, an internal side-chain conformational energy term, and an electrostatic term (Table 1) [13].

For particular targets, a variety of RosettaDock sampling strategies are often used to improve the chance of achieving an accurate structure prediction [28]. If no prior structural or biochemical information is known about the protein interaction of interest, global docking is used to randomize the initial docking poses. From there, score filters and clustering are used to identify clusters of acceptable low-energy structures for further docking and refinement. In most cases, there is some known information about the complex, either in the form of related protein complexes or in biochemical or bioinformatics data which identify probable regions of interaction on the protein partners. In these cases users manually arrange the starting docking pose to a configuration that is compatible with the information and carry out a local docking perturbation. Additionally, users can set distance-based filters that bias sampling towards those docking poses that are compatible with specified constraints [28]. If backbone conformational changes are anticipated, appropriate backbone sampling strategies are prescribed [6], [8], [14], [15].

### Additional capabilities of Rosetta v3.2

Rosetta v3.2 represents a complete bottom-up re-implementation of the Rosetta software. The protein docking module of Rosetta v3.2 was intended to reproduce the core docking functionality of RosettaDock v2.3, and it can be used in conjunction with a number of new Rosetta v3.2 capabilities both through changes in source code and command-line options. These capabilities include automated parameterization of non-protein moieties such as small molecules [25] as well as non-canonical amino acids and post-translational modifications, precise control over the degrees of freedom available during conformational search using the pose, fold tree, and movemap functionalities,[6] expanded side-chain packing options for side-chain optimization and design, and control over sampling and decoy generation through constraints or filters. Finally, a number of algorithmic improvements in sampling and score calculations have led to an overall speed-up. For more information on the capabilities of Rosetta v3.2, see Leaver-Fay et al.[21]

### Implementation of docking in Rosetta v3.2

**Figure 2:**
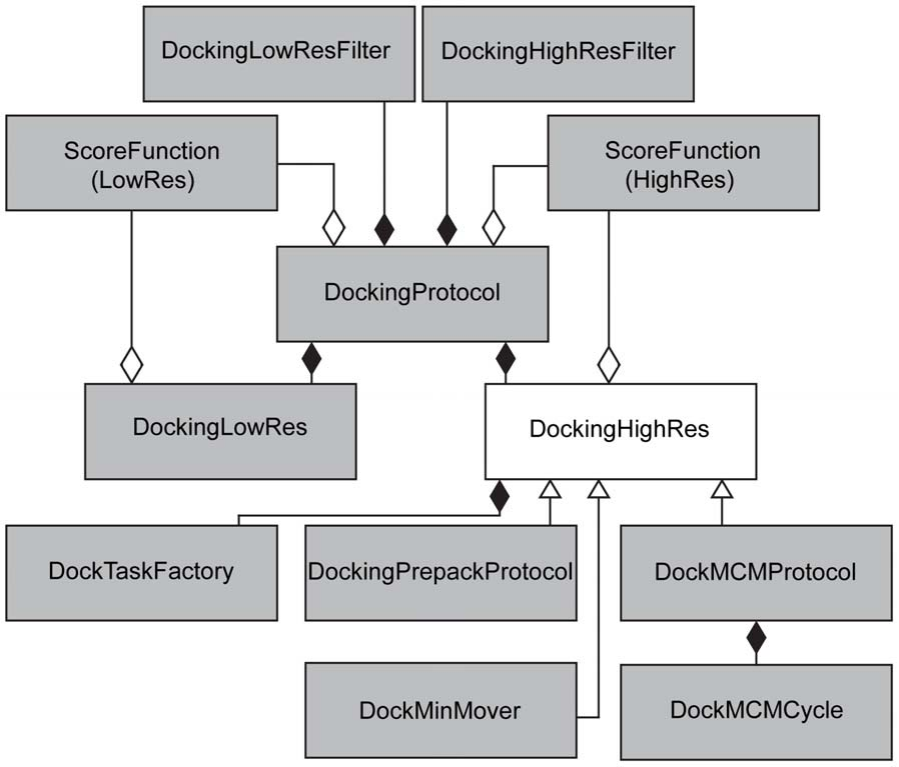
Structure of major classes associated with docking. A shaded diamond indicates composition (the object the diamond points towards is responsible for the lifecycle of the other object); an open diamond indicates aggregation (the object the diamond points towards has an instance of the other object but it may not be solely responsible for that instance's lifecycle); and an open triangle indicates a class hierarchy with the triangle pointing towards the parent class.

The RosettaDock code was restructured in Rosetta v3.2 with two goals: first, to allow for easier use of built-in Rosetta functionality, such as constraints or ligand modeling, and second to give developers greater flexibility when developing their own protocols that use docking functions. Figure 2 shows a diagram of the structure of the major classes associated with docking. Docking has been split into three major classes: DockingProtocol, DockingLowRes and DockingHighRes. DockingProtocol is responsible for handling user-specified docking-options, appropriately configuring various objected associated with docking, and applying DockingLowRes and DockingHighRes objects.

DockingLowRes and DockingHighRes contain all the data and functions associated with the low-resolution docking and high-resolution refinement stages, respectively, including the score functions, sampling functions (including translation/rotation parameters and side-chain packing), and Monte Carlo data. Both objects are independent of the Rosetta options system and can be called directly within the Rosetta source code or through Rosetta interfaces such as PyRosetta[23] and RosettaScripts[22]. Given the wide range of minimization and side-chain packing strategies that might be utilized in the high-resolution docking stage, DockingHighRes is designed as an abstract class that underlies a diverse set of high-resolution docking functions including standard high-resolution docking, pre-packing, as well as extensions of docking such as peptide docking and protein interface design. This versatility is achieved through the DockTaskFactory class within DockingHighRes, which handles all docking side-chain packing options and allows subclasses of DockingHighRes to be able to create a tailored set of packing instructions (Figure 2). All docking objects contain default parameters that allow them to be run with minimal setup; users only need to specify docking parameters for non-default behavior.

### Benchmarking Rosetta with Protein Docking Benchmark 3.0

The expanded Protein Docking Benchmark 3.0, curated by Huang *et al*., [26] is a non-redundant set of bound protein complex structures and their respective unbound structures from the Protein Data Bank.[29] There are 35 complexes classified as ‘enzyme-inhibitor’ (E/I), 25 classified as ‘antibody-antigen’ (Ab/Ag), and 64 classified as ‘other’ (O). According to docking difficulty criteria described by Mintseris *et al*., 88 are ‘rigid-body’ targets, 19 are ‘medium’ targets, and 17 are ‘difficult’ targets. [30] We applied RosettaDock v2.3 and RosettaDock v3.2 to the entire benchmark set.

To prepare the structures for docking, the unbound structures were superimposed over the bound complex and the resulting superposed structure was used as the starting structure for local docking. For consistency, all chain identifiers were switched to that of the bound structure and all hetero-atoms were denoted with a chain identifier of ‘X’. In RosettaDock v2.3 the docking partners are defined by placing a ‘TER’ in the appropriate position in the starting structure PDB file. In RosettaDock v3.2, the docking partners are identified by chain identifier in the command-line.

We first prepared each docking partner in isolation, optimizing their side-chain conformations prior to docking (“pre-packing”),[13] and then carried out local docking perturbations [13] using both RosettaDock v2.3 and RosettaDock v3.2 to generate 1000 decoys, or candidate structures, from each method. As described in [14], we set the docking perturbation parameter to 3 Å translation and 8° rotation. For side-chain packing, extra rotamers were used for χ_1_ for all residues and for χ_2_ for aromatic residues, and unbound rotamers were included as well [27]. We assessed docking performance by sorting these respective decoy sets by interface energy [6]. We ran all docking simulations on our local cluster; creation of each decoy required, on average, 2.5 minutes in RosettaDock v2.3 and 0.8 minutes on RosettaDock v3.2.

The RosettaDock v3.2 command line used for pre-packing and docking structures in this benchmark is shown below. The inputs for docking include the pre-packed starting structure ($name.prepack.pdb), the original starting structure for loading unbound rotamer conformations ($name.unboundrot.pdb), the chain designations for the first and second partner ($chains1 and $chains2), and the desired number of decoys ($nstruct). The starting structure was the unbound components superimposed on the bound complex structure. The output includes all decoys in PDB format, and a score file that includes the total energy (total score), interface energy (I_sc) for each decoy, as well as docking metrics to the starting structure, including Lrmsd (rms), Irmsd (Irms), and *f*_nat_ (Fnat).

#for pre-packing

docking_protocol.linxgccrelease –database $database_path /

-s $name.pdb /

-partners $chains1_$chains2 /

-dock_ppk /

-ex1 -ex2aro -unboundrot $name.unboundrot.pdb

#for docking

docking_protocol.linxgccrelease –database $database_path /

-s $name.prepack.pdb /

–nstruct $nstruct /

-partners $chains1_$chains2 /

-dock_pert 3 8 –spin /

-ex1 -ex2aro -use_input_sc -unboundrot $name.unboundrot.pdb

#for refinement of native complexes

docking_protocol.linxgccrelease –database $database_path /

-s $name.prepack.pdb /

–nstruct $nstruct /

-partners $chains1_$chains2 /

-docking_local_refine /

-ex1 -ex2aro -use_input_sc -unboundrot $name.unboundrot.pdb

For analysis and classification of docking failures we refined native crystal co-complexes using the docking flag –docking_local_refine which carries out only the high-resolution refinement stage of docking. When incorporating non-protein moieties such as ligands or co-factors, additional parameters are input using the flags –extra_res_fa and –extra_res_cen to load the full-atom and centroid mode ligand connectivity and atom typing. We used the script *molfile_to_params.pl* packaged with Rosetta to generate the ligand parameters.[25]

### Metrics for structural accuracy and docking performance

A number of measurements of structural accuracy are regularly used to measure docking performance, as defined by the CAPRI evaluators.[31] L_rmsd is the root mean squared deviation (RMSD) of the C_α_ atoms of the smaller partner in the complex (ligand) to its coordinates in bound structure after superposition of the larger partner in the complex (receptor). I_rmsd is defined as the RMSD of the heavy atoms in the interface residues after superposition of those same residues where the interface is defined as all residues with an intermolecular distance of at most 4.0 Å. Finally, the fraction of native contacts (*f*_nat_) is defined as the fraction of residue-residue contacts in the bound structure that are recovered in a given decoy, where a contact is defined by any two residues with any pair of atoms that are within 5.0 Å. L_rmsd describes the overall ligand-receptor position, I_rmsd describes the atomic accuracy of the interface between the two partners, and *f*_nat_ describes the degree to which specific residue-residue interactions across the interface are recovered. As a qualitative description of accuracy, we use the term ‘near-native’ to refer to a decoy with I_rmsd of at most 4.0 Å.

The presence of a ‘docking funnel’, in which near-native decoys consistently have better scores than non-native decoys, is considered to be the most robust measure of success in a docking simulation. For each target we count the number of near-native decoys among the top five scorers (N_5_) and classify a docking result as having a funnel if it has N_5_≥3.

### Classification of successes and failures

Analyzing docking successes and failures is critical to understanding the strengths and weaknesses of the docking algorithm. Typically structure prediction algorithms are evaluated on the basis of sampling, discrimination, and prediction accuracy. We classified our docking results as successes or failures based on whether they achieved a docking funnel. The quality of a docking success was classified according to the accuracy of the closest decoy in the top five scoring decoys as high, medium, and acceptable quality according to CAPRI-defined criteria.[32] Generally, a decoy with I_rmsd<1.0 Å was considered high quality, 1.0 Å<I_rmsd<2.0 Å was considered medium quality, and 2.0 Å<I_rmsd<4.0 Å, was considered acceptable quality.

For each docking failure we performed two subsequent docking runs. First, we carried out local refinement starting from the unbound partners on the native complex. Second, we carried out local refinement using the bound in their native complex. Based on the results of these subsequent docking runs we classified failures as rigid-body sampling failures (RB sampling failure), backbone-dependent sampling failures (BB sampling failure), and discrimination failure, as described in the Results section.

### Assessing the reliability of docking results: a statistical analysis

In order to quantify the level of variation in the results inherent in a given decoy set, we carried out a bootstrap case resampling.[33] Bootstrap statistical analyses are model-independent and can approximate statistic variables such as mean, standard deviation, and test statistics without making assumptions about the distribution of the underlying data. The noise in inherent in stochastic simulations, the complexity of the conformational landscape that is simulated, and the non-linearity of observed metrics such as RMSD from a reference structure or the number of top-scoring decoys below a given RMSD threshold, necessitate the use of a model-independent statistical approach.

For each target, we generated a set of resampled decoy sets and calculated bootstrap statistical measures based on the observed docking results from each resample. Briefly, we generated *B* resampled decoy sets by randomly selecting 1000 decoys from the original decoy set, with replacement. From this set of re-sampled decoy sets we calculated the bootstrap mean (Eq 1) and standard deviation (Eq 2) of N_5_, denoted µ(N_5_) and σ(N_5_), respectively, based on the observed N_5_ for each re-sample 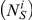. Additionally, we calculated the bootstrap probability of observing a successful docking result based on the number of re-sampled sets where N_5_≥3 (Eq 3). 
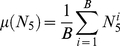
(1)

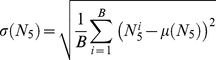
(2)

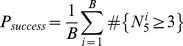
(3)


In order to calculate the significance of a given docking result, we calculated the probability of achieving a docking success by chance. As before, we generated *B* re-sampled decoy sets by randomly selected 1000 decoys from the original decoy set, with replacement, for each target. For each resample, the score for each decoy was replaced by a second random selection of scores from the original decoy set, again with replacement. This second re-sampling randomizes the relationship between a given decoy and its score from docking, while maintaining the overall distribution of decoys and scores, creating a ‘random’ data set specific to each target. We then calculated the above bootstrap statistics 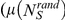, 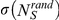, and 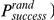 for the randomized data.

## Results

### Overall benchmark results

We applied RosettaDock v3.2 to the Docking Benchmark 3.0, which contains a range of docking targets that vary in both complex type and difficulty. We defined a ‘successful’ prediction as a docking run in which at least three of the five lowest energy structures had an I_rmsd of at most 4.0 Å (N_5_≥3). We defined the quality of a given successful structure prediction as the best accuracy achieved among the five top scoring structures, based on the criteria established in CAPRI for high, medium, and acceptable accuracy.[32] Below we outline the docking results across the entire benchmark set in terms of both its success and reliability in predicting near-native solutions, as well as the overall accuracy of the structure prediction.

**Figure 3:**
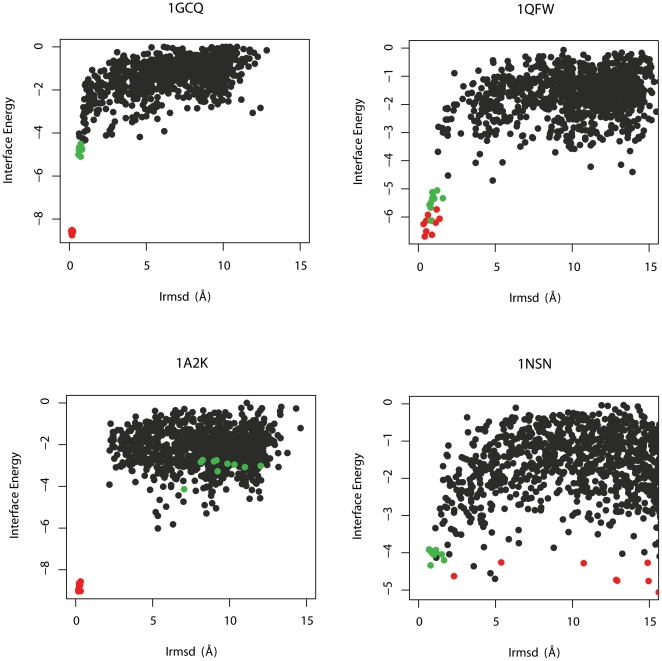
Examples of docking successes and failures. Interface energy vs. I_rmsd scatter plots for representative cases of (A) a docking success, (B) RB-sampling failure, (C) BB-sampling failure, and (D) a discrimination failure. Standard docking run decoys are in gray, the ten lowest-energy decoys from refinement of the unbound conformers superimposed on the native complex are in green, and the ten lowest-energy decoys from refinement of the bound complex is in red.

In a successful docking run, the lowest energy decoys should correspond to the near-native conformations, and at least three of the five top scoring decoys have an Irmsd of ≤4.0 Å. The results of a representative docking success of the Vav-Grb2 complex (1GCQ), is shown as a scatterplot of interface energy vs. Irmsd (Figure 3A). In this example, all five of the five lowest energy decoys are near-native, with the closest having an Irmsd of 0.88 Å, indicating a high-quality prediction. As a comparison, a subsequent refinement of the unbound conformers superimposed on the bound complex (green) identifies similar near-native structures with similar energy, indicating that there was adequate sampling in the standard docking run. A refinement of the bound conformers superimposed on the bound complex (red) demonstrates that the binding-induced conformation changes observed in the Vav-Grb2 co-crystal lead to significantly more favorable interface energies than rigid-body docking of the unbound conformers.

**Figure 4:**
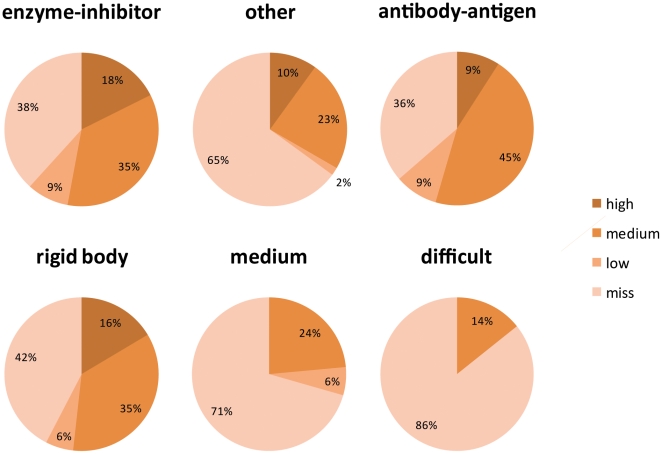
Breakdown of benchmark results. The RosettaDock benchmark performance in terms of docking success and accuracy across both complex type (A) and docking difficulty (B).

The overall benchmark results are illustrated in Figure 4 and summarized in Table 2 and Table 3. Across the 116 targets in the benchmark set, Rosetta v3.2 successfully predicted at least ‘acceptable’ or better quality solutions for 56 targets, representing an overall success rate of 48%. With respect to complex type, Rosetta successfully predicted near-native structures for 62% of the enzyme-inhibitor complexes, 64% of the antibody-antigen complexes, and 35% of the ‘other’ complexes. With respect to docking difficulty, Rosetta successfully predicted near-native structures for 58% of rigid-body targets, 29% of medium difficulty targets, and 14% of difficult targets. Figure 4 provides a further breakdown of docking success and accuracy with respect to both complex type and docking difficulty. Briefly, Rosetta achieved either high or medium accuracy predictions for over 50% for both the enzyme-inhibitor targets and antibody-antigen targets, and 33% of the ‘other’ targets. In terms of docking difficulty, Rosetta achieved either high or medium accuracy for over 50% for rigid-body targets, and almost 25% for medium-difficulty targets, compared to 14% for difficult targets. A deeper analysis reveals that both complex type and docking difficulty were predictors of docking success. Among rigid-body targets, Rosetta predicted 67% of enzyme-inhibitor complexes to at least medium accuracy compared to 52% of antibody-antigen complexes and 40% of ‘other’ complexes.

**Table 2:** Docking Benchmark 3.0 results summary for successes.

PDB	Difficulty | type	N_5_	µ(N_5_) [σ(N_5_)]	*P*_success_	Irmsd	CAPRI quality	PDB	Difficulty | type	N_5_	µ (N_5_) [σ(N_5_)]	*P*_success_	Irmsd	CAPRI quality
1OPH	rigid-body | E	5	5.0 [0.0]	1.00	0.23	***	1YVB	rigid-body | E	4	4.0 [0.9]	0.71	1.32	**
1ML0	rigid-body | O	5	5.0 [0.0]	1.00	0.40	***	1ZHI	rigid-body | O	4	4.0 [0.9]	0.72	1.47	**
1KTZ	rigid-body | O	5	5.0 [0.0]	1.00	0.51	***	1XQS	medium | O	4	3.9 [1.0]	0.70	1.47	**
1PPE	rigid-body | E	5	5.0 [0.0]	1.00	0.91	***	2OOB	rigid-body | O	4	3.9 [1.2]	0.69	1.04	**
1B6C	rigid-body | O	5	5.0 [0.0]	1.00	1.51	**	1DFJ	rigid-body | E	4	3.6 [1.4]	0.57	1.39	**
2HLE	rigid-body | O	5	5.0 [0.2]	1.00	0.89	***	1BJ1	rigid-body | AB	4	3.6 [1.2]	0.57	2.25	*
1KXP	rigid-body | O	5	5.0 [0.2]	1.00	1.16	**	2CFH	medium | O	4	3.6 [1.1]	0.56	1.25	**
2HRK	medium | O	5	5.0 [0.2]	0.99	1.42	**	1BVK	rigid-body | A	3	3.5 [1.2]	0.51	1.77	**
1QA9	rigid-body | O	5	5.0 [0.1]	1.00	0.59	***	1AVX	rigid-body | E	3	3.4 [1.1]	0.50	1.87	**
1FSK	rigid-body | AB	5	5.0 [0.1]	1.00	1.03	**	1MAH	rigid-body | E	3	3.4 [1.1]	0.50	1.94	**
1JPS	rigid-body | A	5	4.9 [0.5]	0.97	1.15	**	1VFB	rigid-body | A	4	3.4 [1.1]	0.50	1.96	**
1AK4	rigid-body | O	5	4.9 [0.5]	0.97	1.36	**	2SNI	rigid-body | E	3	3.3 [1.1]	0.47	1.14	**
1UDI	rigid-body | E	5	4.9 [0.4]	0.98	2.17	*	1KXQ	rigid-body | AB	3	3.3 [1.1]	0.44	1.25	**
1D6R	rigid-body | E	5	4.9 [0.3]	0.99	2.14	*	1BUH	rigid-body | O	3	3.3 [1.1]	0.44	1.73	**
7CEI	rigid-body | E	5	4.8 [0.6]	0.94	0.79	***	1XD3	rigid-body | O	3	3.3 [1.1]	0.45	2.69	*
2UUY	rigid-body | E	5	4.7 [0.7]	0.93	1.30	**	1E4K	difficult | A	4	3.2 [1.3]	0.45	1.98	**
1E6E	rigid-body | E	5	4.7 [0.6]	0.94	0.79	***	1E6J	rigid-body | A	3	3.2 [1.2]	0.40	2.48	*
1SBB	rigid-body | O	5	4.6 [0.7]	0.91	0.60	***	1HIA	rigid-body | E	3	3.2 [1.1]	0.40	1.95	**
2C0L	difficult | O	5	4.6 [0.7]	0.91	1.15	**	2SIC	rigid-body | E	3	3.1 [1.3]	0.40	0.59	***
1IQD	rigid-body | AB	5	4.5 [0.8]	0.89	1.26	**	2FD6	rigid-body | A	3	3.1 [1.2]	0.38	1.85	**
1AHW	rigid-body | A	5	4.5 [0.7]	0.89	1.38	**	1HE1	rigid-body | O	3	3.0 [1.2]	0.36	1.31	**
1GCQ	rigid-body | O	5	4.4 [0.8]	0.88	0.72	***	2JEL	rigid-body | AB	3	3.0 [1.1]	0.36	0.40	***
1EAW	rigid-body | E	4	4.4 [0.8]	0.87	1.31	**	1AY7	rigid-body | E	3	2.9 [1.1]	0.32	1.55	**
1FC2	rigid-body | O	4	4.4 [0.8]	0.84	1.53	**	1WQ1	medium | O	3	2.8 [1.3]	0.30	1.48	**
1GPW	rigid-body | O	4	4.4 [0.8]	0.88	1.98	**	2QFW	rigid-body | AB	3	2.8 [1.2]	0.30	0.64	***
2MTA	rigid-body | E	4	4.3 [0.9]	0.81	0.66	***	1IJK	medium | E	3	2.8 [1.2]	0.28	2.35	*
1BVN	rigid-body | E	4	4.0 [1.0]	0.72	1.35	**	1NCA	rigid-body | AB	3	2.7 [1.3]	0.27	0.46	***
1CGI	rigid-body | E	4	4.0 [1.0]	0.74	1.76	**	2I25	rigid-body | A	3	2.2 [1.2]	0.15	1.80	**

**Table 3:** Docking Benchmark 3.0 results summary for failures.

PDB	Difficulty | type	N_5_	µ (N_5_) [σ(N_5_)]	*P*_success_	Irmsd	Classification	PDB	Difficulty | type	N_5_	µ (N_5_) [σ(N_5_)]	*P*_success_	Irmsd	Classification
1K74	rigid-body | O	2	2.5 [1.1]	0.20	1.63	RB sampling	1EER	difficult | O	1	0.9 [1.0]	0.02	2.23	BB sampling
1Z5Y	rigid-body | O	2	2.4 [1.1]	0.16	1.14	BB sampling	1FAK	difficult | O	1	0.9 [0.9]	0.01	3.30	discrimination
1WEJ	rigid-body | A	2	2.2 [1.1]	0.14	2.60	discrimination	1TMQ	rigid-body | E	1	0.8 [0.9]	0.01	1.47	RB sampling
1HE8	medium | O	2	2.2 [1.1]	0.13	3.19	BB sampling	1I4D	rigid-body | O	1	0.8 [0.9]	0.01	3.13	RB sampling
1ATN	difficult | O	2	2.1 [1.2]	0.12	1.53	RB sampling	1RLB	rigid-body | O	1	0.8 [0.9]	0.01	3.88	RB sampling
2HQS	rigid-body | O	2	2.1 [1.2]	0.13	2.69	BB sampling	1Z0K	rigid-body | O	1	0.7 [0.8]	0.00	3.56	BB sampling
1JMO	difficult | O	2	2.1 [1.2]	0.11	2.82	BB sampling	1EFN	rigid-body | O	1	0.7 [0.8]	0.00	3.58	BB sampling
2O8V	rigid-body | E	2	2.1 [1.2]	0.12	3.62	BB sampling	1I9R	rigid-body | AB	1	0.6 [0.8]	0.00	0.52	RB sampling
1J2J	rigid-body | O	2	2.0 [1.4]	0.14	1.10	BB sampling	1AZS	rigid-body | O	0	0.5 [0.9]	0.01	5.41	RB sampling
1NSN	rigid-body | AB	2	2.0 [1.2]	0.10	1.15	discrimination	2B42	rigid-body | E	0	0.4 [0.6]	0.00	5.85	RB sampling
1K5D	medium | O	2	2.0 [1.1]	0.09	2.87	BB sampling	2BTF	rigid-body | O	0	0.3 [0.6]	0.00	4.44	BB sampling
1FQ1	difficult | E	2	1.7 [1.3]	0.08	2.18	RB sampling	1KKL	medium | E	0	0.2 [0.5]	0.00	4.60	RB sampling
2AJF	rigid-body | O	2	1.7 [1.1]	0.06	1.23	RB sampling	1F51	rigid-body | O	0	0.2 [0.5]	0.00	12.35	BB sampling
1E96	rigid-body | O	1	1.6 [1.1]	0.05	3.38	BB sampling	1KAC	rigid-body | O	0	0.1 [0.5]	0.00	4.01	BB sampling
1I2M	medium | O	1	1.5 [1.0]	0.03	2.68	BB sampling	1MLC	rigid-body | A	0	0.1 [0.4]	0.00	4.31	BB sampling
1QFW	rigid-body | AB	1	1.5 [1.1]	0.03	1.93	RB sampling	1S1Q	rigid-body | O	0	0.1 [0.3]	0.00	4.37	BB sampling
1GLA	rigid-body | O	2	1.5 [1.1]	0.05	2.98	BB sampling	1M10	medium | E	0	0.0 [0.0]	0.00	4.46	BB sampling
1AKJ	rigid-body | O	1	1.2 [1.0]	0.02	1.48	BB sampling	1A2K	rigid-body | O	0	0.0 [0.0]	0.00	5.20	BB sampling
1EZU	rigid-body | E	1	1.2 [1.0]	0.02	2.82	RB sampling	1R8S	difficult | O	0	0.0 [0.0]	0.00	5.21	BB sampling
1F34	rigid-body | E	1	1.2 [1.0]	0.02	4.00	RB sampling	1GP2	medium | O	0	0.0 [0.0]	0.00	5.23	BB sampling
1GRN	medium | O	1	1.1 [1.0]	0.02	1.24	BB sampling	1IBR	difficult | O	0	0.0 [0.0]	0.00	5.46	RB sampling
1NW9	medium | E	1	1.1 [1.0]	0.02	2.33	BB sampling	1BKD	difficult | O	0	0.0 [0.0]	0.00	5.62	BB sampling
2H7V	medium | O	1	1.1 [1.0]	0.02	3.65	BB sampling	2OT3	difficult | O	0	0.0 [0.0]	0.00	6.00	BB sampling
1GHQ	rigid-body | O	1	1.0 [1.0]	0.01	2.69	BB sampling	1FQJ	rigid-body | O	0	0.0 [0.0]	0.00	6.48	RB sampling
2NZ8	medium | O	1	1.0 [1.0]	0.02	3.91	BB sampling	1PXV	difficult | E	0	0.0 [0.0]	0.00	9.49	BB sampling
1IB1	medium | O	1	1.0 [1.0]	0.01	3.95	discrimination	1H1V	difficult | O	0	0.0 [0.0]	0.00	9.96	BB sampling
1K4C	rigid-body | AB	1	1.0 [1.1]	0.02	2.15	BB sampling	1Y64	difficult | O	0	0.0 [0.0]	0.00	13.70	discrimination
1EWY	rigid-body | E	1	1.0 [1.1]	0.02	3.01	BB sampling	1DQJ	rigid-body | A	0	0.0 [0.2]	0.00	6.15	BB sampling
2PCC	rigid-body | E	1	1.0 [0.9]	0.01	3.38	discrimination	1ACB	medium | E	0	0.0 [0.1]	0.00	4.56	RB sampling
							1KLU	rigid-body | O	0	0.0 [0.1]	0.00	8.16	BB sampling

Unsuccessful docking predictions are classified as cases for which the five lowest-energy decoys did not contain at least three near-native structures. There are a number of potential reasons for docking failures, from insufficient sampling of near-native conformations to inadequate discrimination of near-native structures from the overall decoy set. We sought to classify the observed docking failures based on subsequent docking refinement runs that identify whether inadequate rigid-body sampling, binding-induced conformation changes, or deficiencies in the score function are responsible for failure.

Docking failures due to inadequate rigid-body sampling (RB sampling failure) reflect cases where rigid-body docking of the unbound conformers is sufficient for successful docking, but rigid-body conformational space was insufficiently sampled to locate low-energy near-native structures in the standard docking run. The Fv antibody-human chorionic gonadtropin complex (1QFW) serves representative example of an RB sampling failure (Figure 3B). A scatter-plot of interface energy vs. Irmsd shows that refinement of the unbound conformers superimposed on the bound complex located lower energy near-native decoys than those accessed by the standard docking run.

Docking failures due to binding-induced conformation changes between the unbound and bound state (BB sampling failures) arise from cases in which rigid-body docking of the unbound conformers is incapable of identifying low-energy near-native conformations. In such cases, binding-induced conformation changes must be taken into account for successful docking. The nuclear transport factor 2-Ran GTPase complex (1A2K) serves as a representative example of a BB sampling failure (Figure 3C). Neither standard docking nor refinement of the unbound conformers superimposed on the bound complex were capable of sampling low-energy near-native conformations. By contrast, refinement of the bound conformers superimposed on the bound complex locates near-native structures with significantly more favorable interface energy than the decoys from unbound docking.

Finally, discrimination failures arise from cases where even refinement of the bound conformers superimposed on the bound complex are unable to identify lower energy decoys than those accessed by the standard docking runs. The N10 antibody-staphylococcal nuclease complex (1NSN) illustrates a discrimination failure (Figure 3D). Regardless of whether the bound or unbound conformation is used, there is no discrimination of near-native decoys.

Overall, among the 59 targets in which RosettaDock failed, 17 were considered RB-sampling failures, 36 were considered BB-sampling failures, and 6 were considered discrimination failures. There is a strong relationship between both docking difficulty and complex type with regards to the cause of docking failure (Table 4). Most notably, docking of medium and difficult targets resulted in BB-sampling failures over 50% of the time. Likewise, 45% of ‘other’ type complexes resulted in BB-sampling failures.

**Table 4:** Docking success and failure by complex type and difficulty.

				failures % [n]	
complex type	*n*	Success % [n]	RB sampling	BB sampling	discrimination
antibody-antigen	23	67 [16]	9 [2]	14 [3]	9 [2]
enzyme-inhibitor	33	61 [20]	21 [7]	15 [5]	3 [1]
other	60	35 [21]	15 [9]	45 [27]	5 [3]
**difficulty**					
rigid-body	84	58 [49]	15 [13]	23 [19]	4 [3]
medium	17	29 [5]	12 [2]	53 [9]	6 [1]
difficult	14	14 [2]	21 [3]	50 [7]	14 [2]

### Measuring variability in docking results

Given that a number of docking programs, including RosettaDock, are based on stochastic sampling strategies, an understudied area of research is in quantifying the degree of certainty of a given docking prediction. We used a bootstrap case resampling approach to quantify the variability within a given docking run. We found that 5000 re-sampled sets (*B* = 5000) for each target was more than sufficient to converge the bootstrap statistical measures of interest: mean N_5_ (µ (N_5_)), standard deviation of N_5_ (σ(N_5_)), and the percentage of re-sampled sets which could be classified as a docking success (*P*_success_). The bootstrap statistics are displayed in Table 2 and 3 for docking successes and failures, respectively.

The µ (N_5_) showed good agreement with the N_5_ calculated, indicating both that our original N_5_ data did not contain any significant outliers, and that the µ (N_5_) metric was well-converged. The inherent noise within each decoy set is quantified by both σ(N_5_) and by *P*_success_. Overall, there was significant noise in the data. In approximately 33% of the successes, *P*_success_ was greater than 0.9, indicating an extremely reliable success rate. For cases closer to the borderline of success classification, *P*_success_ fell below 0.3, implying that in repeated trials, those targets would be considered docking successes only 30% of the time. Among docking failures, few targets had a µ (N_5_) greater than 2.0 or a *P*_success_ greater than 0.15. Using statistics-based docking success criteria of µ (N_5_) ≥2.5 and *P*_success_ ≥0.3, 53 of 56 docking successes remained classified as successes indicating that the overall results of the benchmark are robust to the noise inherent in the stochastic sampling methods used in RosettaDock. Finally, a bootstrap analysis of target-specific randomized re-sampled sets showed that the probability of observing a funnel from randomized data 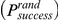 was below 0.001 for all docking targets, demonstrating the significance of observing a docking funnel in the data.

### Comparing RosettaDock v3.2 and v2.3

**Figure 5:**
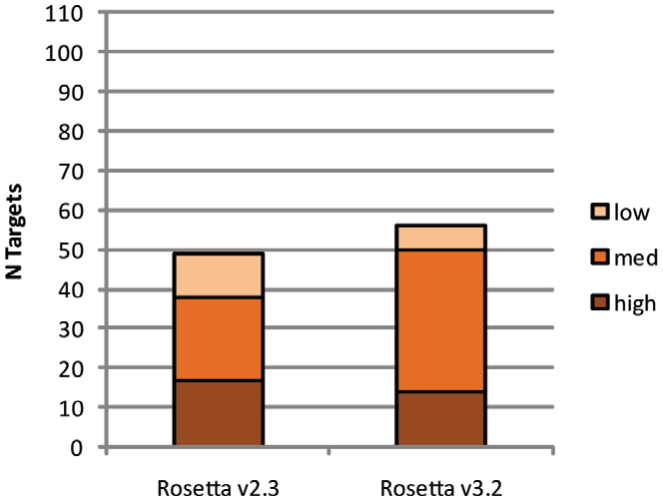
Comparison of RosettaDock v3.2 and RosettaDock v2.3. A histogram showing the docking success and accuracy for a benchmark set of 116 targets for the new RosettaDock v3.2 and the older RosettaDock v2.3.

One major goal in developing the new RosettaDock v3.2 was to reproduce, if not improve, the docking performance and accuracy of the previous RosettaDock v2.3. Towards that end, we ran the entire benchmark set using RosettaDock v2.3 for comparison (Tables S1 and S2). In terms of computational speed, RosettaDock v3.2 substantially outperformed the older version, generating decoys, on average, three times faster. Given that further docking improvements will inevitably require more computational intensive sampling as well as more sophisticated score functions, this speed-up is essential to developing future docking strategies. In terms of docking performance, RosettaDock v3.2 performed marginally better than the older version, despite having the same overall docking algorithm. As shown in the histogram on Figure 5, overall, RosettaDock v3.2 achieved 56 successful predictions, compared to 49 in v2.3. Furthermore, it was more accurate, with 50 predictions of medium or high accuracy, compared with 38.

We calculated bootstrap statistics for RosettaDock v2.3 to compare with the v3.2. The bootstrap analysis revealed that the differences observed in the overall number of successes and failures classified by the N_5_ measure was supported by significant differences in the underlying data. Among 58 targets in which RosettaDock v2.3 *or* RosettaDock v3.2 produced a *P*_success_≥0.30, there were 13 targets where v3.2 showed at least a 5-fold increase *P*_success_ relative to v2.3 compared to 3 targets in which v2.3 showed similar improvement over v3.2. A visual inspection of these targets shows that *P*_success_ quantifies readily observable qualitative differences between these docking results well.

### Docking with ligand groups

**Figure 6:**
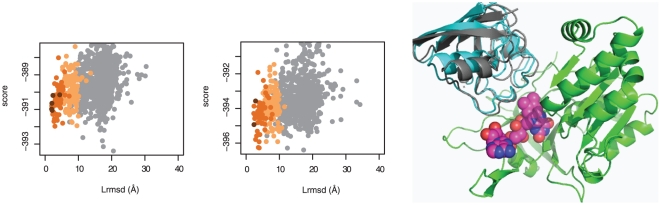
Docking of the FNR-Fn ternary complex. Plots of score vs. Lrmsd for local docking of the unbound structures in target 1EWY without (A) and with (B) the small molecule FAD bound to FNR (A), with high, medium, and acceptable accuracy decoys colored in brown, orange, and tan, respectively. (C) The second-lowest energy structure from docking using FAD with FNR (green), Fd (cyan), and the FAD molecule (magenta) superimposed on the crystal structure of the complex (gray).

We used the small-molecule modeling capabilities of Rosetta v3.2 to carry out protein docking on a subset of six targets in the benchmark where a small molecule was found in or near the interface. The small molecule was explicitly modeled with all atoms, including hydrogens, in both the low-resolution and high-resolution stages of docking. The Ferredoxin-NADP+ reductase (FNR) bound to the ligand group flavin adenine dinucleotide (FAD), in complex with ferredoxin (Fd) (Target 1EWY) provides the best example of the potential benefits to explicitly modeling ligands in protein docking. Figure 6 shows the score vs. I_rmsd_ plot for docking with and without modeling the FAD molecule, and the second-lowest-energy decoy illustrated. The FAD molecule serves as a prosthetic group in FNR and is critical for electron transfer from the Fd to the NADP+ substrate through the formation of a ternary complex.[34] Inspection of the crystal structure of the complex shows that almost half of the intermolecular contacts between FNR and Fd are mediated by the FAD molecule.[35] The necessity of explicitly modeling the FAD molecule in protein docking of this target highlights its critical role in mediating the formation of this protein complex and underscores the need to accommodate interface ligands and cofactors in protein docking.

Overall, among the six targets tested, explicitly modeling the small molecule at the interface substantially improved docking in three cases, and did not have any effect for the other four (Table 5). In all the targets except 1EWY, interface small molecules made up only a very small fraction of the total intermolecular contacts at the interface. The greatest improvements were observed in targets 1EWY and 1GRN, where local docking without the small-molecules failed to produce even a single medium-quality prediction in the five top scoring decoys. Bootstrap analysis of docking results showed that in three cases, 1EWY, 1GRN, and 1RLB, docking with the ligand lead to a ∼10 fold improvement in *P*_success_, indicating improvements in docking that aren't captured by the final N_5_ metric. It is important to note that protein docking with explicitly modeled ligands might require a re-tuned score function, as there are a number of differences between the protein docking and ligand docking scoring function in Rosetta, mainly in the balance of hydrogen bonding and pair potential terms with respect to the electrostatics and solvation terms.

**Table 5:** Results of incorporating small molecules in Rosetta v3.2.

		without ligand				with ligand			
PDB	ligand	N_5_	µ (N_5_) [σ(N_5_)]	P_success_	I_rmsd_ (Å)	N_5_	µ (N_5_) [σ(N_5_)]	P_success_	I_rmsd_ (Å)
1EWY	FAD	1	1.0 [1.1]	0.02	3.01	3	2.6 [1.2]	0.22	1.07
1GRN	GDP, Mg	1	1.1 [1.0]	0.02	1.24	2	2.3 [1.2]	0.18	1.02
1WQ1	GDP, Mg	3	2.8 [1.3]	0.30	1.48	2	1.9 [1.2]	0.12	2.90
1RLB	Retinoic acid	1	0.8 [0.9]	0.01	3.88	2	2.1 [1.3]	0.16	1.99
1R8S	GDP	0	0.0 [0.0]	0.00	5.21	0	0.0 [0.0]	0.0	4.19

## Discussion

In this study we benchmarked the docking performance of the new RosettaDock v3.2 against the diverse Protein Docking Benchmark 3.0, marking the most comprehensive benchmarking of the RosettaDock algorithm. Our goals were two-fold: to identify trends in the docking results that may serve as areas of improvement in the algorithm development and to benchmark the new RosettaDock version against the popular previous iteration of RosettaDock, v2.3. Finally, we aimed to showcase the capabilities of the Rosetta v3.2 modeling package by incorporating small-molecule and ligand cofactors in the docking simulation, a capability that is lacking in the earlier RosettaDock as well as many other protein docking protocols.

Overall RosettaDock v3.2 achieved a 48% success rate over the entire benchmark set with substantial variation in results across both complex type and docking difficulty. The most consistently accurate predictions came for enzyme-inhibitor and antibody-antigen complexes, while the results for ‘other’ complexes varied significantly. Docking difficulty, as classified by Mintseris et al. [30], is based on the magnitude of backbone conformational changes between the bound and unbound conformations. Rosetta's docking algorithm performed well with rigid-body targets where side-chain flexibility is adequate in accommodating binding-induced conformational changes. By contrast, medium and difficult targets proved to be a challenge for the standard RosettaDock algorithm.

Evaluations of structure-based algorithms typically treat sampling and discrimination as orthogonal metrics. A cursory survey of the benchmark results shows that despite a success rate of approximately 50%, near-native rigid-body orientations were sampled in over 90% of the targets. This sizeable ‘discrimination gap’ suggests that discrimination of near-native structures is the primary challenge for future docking development. Given that sampling and discrimination are intimately linked when using physics-based sampling and scoring strategies, we sought to more finely distinguish sampling and discrimination by examining docking failures in the context of rigid-body docking, binding-induced backbone conformation changes, and near-native discrimination. We identified three corresponding types of docking failures: RB sampling failure, in which the native structure was at the global energy minimum when docking the unbound conformations but was undersampled in standard docking, BB sampling failure in which the native structure was at the global energy minimum only when docking the bound backbone conformations and thus inaccessible to rigid-body docking using the unbound backbone conformations, and a discrimination failure in which the global energy minimum does not correspond to the native structure.

An analysis of the docking failures revealed significant trends as to the cause of failure. In almost all categories of complex type and difficulty, BB sampling failures were the most common, and accounted for over 60% of all failures. By contrast, true discrimination failures accounted for less than 10% of docking failures, indicating that the apparent ‘discrimination gap’ between the number of successful predictions and the number of targets in which near-natives were sampled is largely due to the sub-optimal backbone conformations of the unbound state.

It is notable that ‘other’ type complexes are particularly prone to the BB sampling failure; even in local docking only 50% of other-type complexes identified the native conformation, compared to 78% and 82% for enzyme-inhibitor and antibody-antigen complexes respectively. This could reflect historical biases towards enzyme-inhibitor targets for which docking algorithms were first developed, or it could reflect more challenging flexibility and thermodynamics in this broad class of complexes. Unlike traditional enzyme-inhibitor complexes which typically have small conformation changes and higher affinities, these ‘other’ complexes are often involved in molecular recognition or signal transduction and bind with greater promiscuity and lower binding affinities. Substantial advancements have been made that expand the core protein docking algorithm in RosettaDock to accommodate binding-induced backbone conformation changes, both through explicitly modeling of backbone flexibility[6] as well as the use of an ensemble of alternate backbone conformers.[14] Once these features have been fully implemented in RosettaDock v3.2, a benchmarking against the data set presented here is needed.

A bootstrapping-based statistics approach to measuring the variability in docking results, such as calculating the probability of observing a docking success, can be a useful tool for comparing stochastic data from structure prediction methods like RosettaDock. Previous statistical descriptions, such as using a z-score based on the mean and standard deviation of data [27] makes f,awed assumptions about the distribution of the underlying data, which can lead to significant noise in the final statistical measure, limiting both its accuracy and utility. Likewise, a visual assessment of prediction quality from the data, while useful in qualitatively evaluating the results in a manner robust to the noise of a particular metric, is subject to user-bias.[36] By contrast, a relatively simple, model-independent, bootstrapping method, such as the case re-sampling approach described here, avoids these pitfalls while describing both the reliability and significance of the structure prediction results. Further research on the utility of bootstrapping statistics to analyze stochastic results is needed, particularly in the context of molecular modeling and structure prediction.

The new Rosetta v3.2 modeling package provides a powerful means for developing novel and customized modeling protocols based on several core algorithms. The RosettaDock algorithm, which searches rigid-body conformation space to optimize the interface between two protein segments continues to be a highly useful molecular modeling tool for a range of applications from protein docking to interface design.

## Supporting Information

Table S1 (XLS)

Table S2 (XLS)
